# Do *Babesia microti* Hosts Share a Blood Group System Gene Ortholog, Which Could Generate an Erythrocyte Antigen That Is Essential for Parasite Invasion?

**DOI:** 10.3390/tropicalmed9090195

**Published:** 2024-08-26

**Authors:** Ryan P. Jajosky, Audrey N. Jajosky, Philip G. Jajosky, Sean R. Stowell

**Affiliations:** 1 Joint Program in Transfusion Medicine, Brigham and Women’s Hospital, Harvard Medical School, 630E New Research Building, 77 Avenue Louis Pasteur, Boston, MA 02115, USA; 2 Biconcavity Inc., Lilburn, GA 30047, USA; 3 Department of Pathology and Laboratory Medicine, University of Rochester Medical Center, Rochester, NY 14586, USA

**Keywords:** parasitology, host tropism, tick-borne illness, bioinformatics, zoonoses, Apicomplexa, global health, host–parasite interactions, transfusion medicine, RBC exchange, therapeutically rational exchange transfusion

## Abstract

The United States of America (US) has the highest annual number of human babesiosis cases caused by *Babesia microti* (*Bm*). *Babesia*, like malaria-causing *Plasmodium,* are protozoan parasites that live within red blood cells (RBCs). Both infectious diseases can be associated with hemolysis and organ damage, which can be fatal. Since babesiosis was made a nationally notifiable condition by the Centers for Disease Control and Prevention (CDC) in January 2011, human cases have increased, and drug-resistant strains have been identified. Both the *Bm* ligand(s) and RBC receptor(s) needed for invasion are unknown, partly because of the difficulty of developing a continuous in vitro culture system. Invasion pathways are relevant for therapies (e.g., RBC exchange) and vaccines. We hypothesize that there is at least one RBC surface antigen that is essential for *Bm* invasion and that all *Bm* hosts express this. Because most RBC surface antigens that impact *Plasmodium* invasion are in human blood group (hBG) systems, which are generated by 51 genes, they were the focus of this study. More than 600 animals with at least one hBG system gene ortholog were identified using the National Center for Biotechnology Information (NCBI) command-line tools. Google Scholar searches were performed to determine which of these animals are susceptible to *Bm* infection. The literature review revealed 28 *Bm* non-human hosts (NHH). For 5/51 (9.8%) hBG system genes (e.g., *RhD*), no NHH had orthologs. This means that RhD is unlikely to be an essential receptor for invasion. For 24/51 (47.1%) hBG system genes, NHH had 4–27 orthologs. For the *ABO* gene, 15/28 NHH had an ortholog, meaning that this gene is also unlikely to generate an RBC antigen, which is essential for *Bm* invasion. Our prior research showed that persons with blood type A, B, AB, O, RhD+, and RhD- can all be infected with *Bm*, supporting our current study’s predictions. For 22/51 (43.1%) hBG system genes, orthologs were found in all 28 NHH. Nineteen (37.3%) of these genes encode RBC surface proteins, meaning they are good candidates for generating a receptor needed for *Bm* invasion. In vitro cultures of *Bm*, experimental *Bm* infection of transgenic mice (e.g., a CD44 KO strain), and analyses of *Bm* patients can reveal further clues as to which RBC antigens may be essential for invasion.

## 1. Introduction

Most human babesiosis cases are in the United States of America (US), and the majority are caused by *Babesia microti* (*Bm*) [1]. In January 2011, the Centers for Disease Control and Prevention (CDC) made babesiosis a nationally notifiable condition [2]. Since then, cases have increased and drug-resistant strains have been identified [3,4,5,6]. *Babesia*, like malaria-causing *Plasmodium,* are intraerythrocytic protozoan parasites, meaning that they live within red blood cells (RBCs). Both infectious diseases can be associated with hemolysis and organ damage, which can be fatal. Most human *Bm* infections are transmitted via the black-legged tick (i.e., deer tick, *Ixodes scapularis*, and, formerly, *Ixodes dammini*). The white-footed mouse (*Peromyscus leucopus*) is often considered to be the most important natural host of *Bm*, yet many other mammals and birds can be infected [7,8]. 

Identifying the *Bm* ligand(s) and RBC receptor(s) that are essential for invasion could lead to the first *Bm*-specific therapy or vaccine. For example, patients could undergo an RBC exchange in which *Bm*-parasitized RBCs are replaced with invasion-resistant RBCs (i.e., therapeutically-rational exchange, T-REX) [9,10,11,12,13,14,15,16,17]. In contrast, there are malaria-specific treatments and vaccines [18,19]. In 1975, an in vitro assay revealed that *Plasmodium knowlesi* (*Pk*) requires ACKR1 (i.e., Duffy) on RBCs to invade [16]. In 1976, researchers found that only persons with ACKR1+ RBCs could be infected with *P. vivax* (*Pv*) [20]. In 1988 and 1989, *Pk* and *Pv* Duffy-binding proteins (DBPs) were identified, respectively [21,22]. *Pk* patients can undergo RBC exchange using invasion-resistant ACKR1- RBCs [16]. The leading *Pv* subunit vaccine candidate is targeted against DBP [23], while the leading one for *Pf* is against Rh5, which binds basigin, an essential reticulocyte/RBC receptor [24]. 

Pathogens can infect different animals if host cells have an ortholog that generates an antigen that is essential for invasion [25]. Orthologs are “genes which evolved from a common ancestral gene by speciation that usually have retained a similar function in different species” [26]. For *Pf*, humans are the natural host, while chimpanzees (*Pan troglodytes*) can also be infected but not gorillas (*Gorilla gorilla*) [27]. Humans and chimpanzees have a *BSG* ortholog, which encodes basigin, and *Pf*Rh5 can bind to both. Although the western lowland gorilla (*Gorilla gorilla gorilla*) shares a *BSG* ortholog with humans and chimpanzees, researchers have shown that *Pf*Rh5 is unable to bind gorilla basigin, likely due to amino acid changes at positions 27, 100, and 103 [27]. 

It was hypothesized that all *Bm* hosts share a gene ortholog that generates a similar RBC surface antigen, which is essential for parasite invasion. Although humans have around 20,000 protein-coding genes [28], the list can be narrowed down by focusing on human blood group (hBG) system genes, of which many are important for *Plasmodium* invasion. Importantly, researchers have shown that *Bm* prefers to invade mouse RBCs more than reticulocytes, justifying the focus on hBG system genes [29]. Ultimately, we used bioinformatics to identify hBG system gene orthologs that are shared by all *Bm* hosts, to narrow down the list of hBG system antigens that may be essential for invasion. 

## 2. Materials and Methods

This study was conducted from January 2023 to May 2024.

### 2.1. hBG System Genes and Orthologs

A list of hBG systems and genes was obtained from the International Society of Blood Transfusion (ISBT) [30]. Each gene was typed into the NCBI Gene database to obtain the NCBI gene ID, gene symbol, and gene name [31]. Then, NCBI Datasets command-line tools were used, instead of the NCBI website, per recommendation from the National Library of Medicine (NLM) Help Desk (personal communication) [32]. The Command Prompt application on a Windows PC was opened. Then, “datasets download gene gene-id 28 --ortholog all --filename orthologs.zip” was entered. (Note: No quotation marks were used and the gene id was changed each time.) Next, “dataformat tsv gene --fields tax-name --package orthologs.zip” was entered. The list of animals with gene orthologs was highlighted, copied, and pasted into a Microsoft Excel spreadsheet for each hBG system gene. Thus, the ortholog calculations were performed by NCBI [33]. The authors did not use BLAST to make ortholog calculations.

### 2.2. Literature Review

Google Scholar searches were performed to find studies looking for *Bm* infection in each animal with an hBG system gene ortholog [34]. The search query consisted of the genus and species or common name with the terms “*Babesia microti*” and “PCR” (i.e., polymerase chain reaction). An example search query was “*Peromyscus leucopus Babesia microti* PCR”. (Note: No quotation marks were used in the query). Some articles referred to the animal by its common name (e.g., dog) instead of its scientific name (e.g., *Canis lupus familiaris*) [35]. Occasionally, an article would use a synonym for the animal’s scientific name in NCBI (*Clethrionomys glareolus* = *Myodes glareolus*, *Papio cynocephalus anubis* = *Papio anubis*) [36,37]. Articles were not excluded for these reasons. 

The original plan was to only include studies that used PCR to detect *Bm* infection in hosts. Some studies described experimental *Bm* infection of animals without the use of PCR [38,39]. Other studies used xenodiagnosis, which warrants explanation. Female *I. scapularis* ticks cannot transmit *Bm* to their offspring (i.e., no transovarial transmission) [40]. A tick egg hatches into a larva, then molts into a nymph, followed by molting into an adult in a 2-year life cycle [41]. Here, xenodiagnosis means allowing an uninfected larva to feed on a captured animal, followed by testing the larva for *Bm* [7,8]. Studies using experimental infection or xenodiagnosis were included in this analysis. In contrast, studies diagnosing natural *Bm* infection using peripheral blood smears were excluded because different *Babesia* species can have similar morphology [42]. Sometimes, nymph or adult ticks on animals were tested for *Bm*, but the host was not. These studies were excluded because some animals (e.g., white-tailed deer, WTD, also known as *Odocoileus virginianus*) can harbor *Bm*-infected ticks but are resistant to *Bm*.

### 2.3. Animal Taxonomy and Total Number of Genes in NCBI

Each *Bm* non-human host (NHH) was typed into NCBI Taxonomy to determine its Genbank common name, NCBI BLAST name, and its class (e.g., Mammalia) [43]. The animal was also typed into NCBI Datasets, and the button “Genes” with the subheading “Browse all” was clicked to determine the total number of genes in NCBI [44]. 

## 3. Results

There are 45 hBG systems, which include 51 genes. More than 600 animals had at least one hBG system gene ortholog (see Supplemental File). Based on the literature review, 28 NHH were identified, all of which are mammals (Table 1). The NCBI reports orthologs for *P. maniculatus bairdii*, yet one study that showed *P. maniculatus* susceptibility to *Bm* did not include the subspecies name [45]. Another study examined *P. maniculatus gracilis*, a different subspecies [46]. Because the meaningfulness of subspecies is controversial, these studies were included in this analysis [47]. 

At least one study performed PCR on NHH tissues to confirm *Bm* infection, except for two NHH. The only two studies showing *Cricetulus griseus* can be infected with *Bm* did not use PCR or molecular testing to confirm infection [38,39]. Researchers used peripheral blood smears to show that *C. griseus* can be infected by *Bm* strain AJ, which was derived from a patient [38,39]. Only two studies found that *Sciurus carolinensis* can be infected by *Bm* and these studies used xenodiagnosis instead of PCR of the animal’s tissues. 

**Figure 1:**
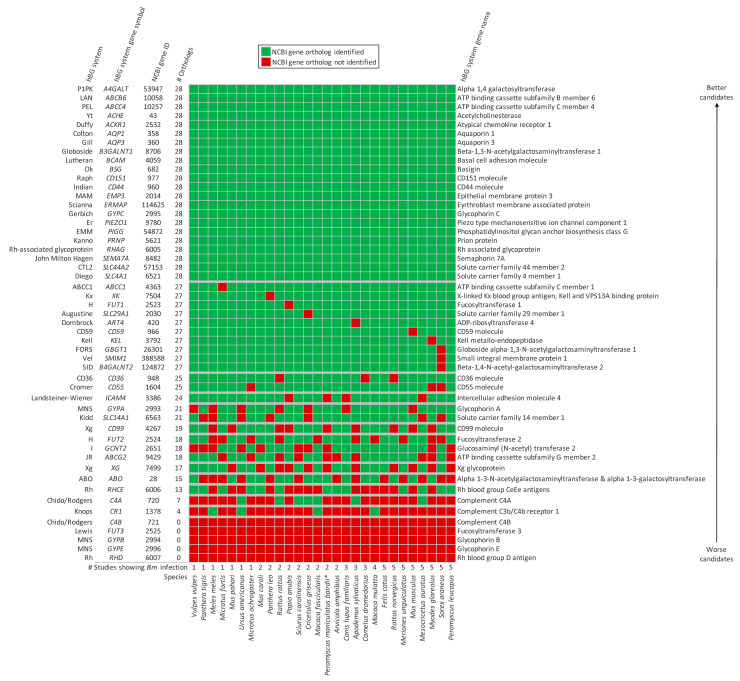
The presence or absence of hBG system gene orthologs in 28 *Bm* NHH. Genes for which all 28 NHH have an ortholog are considered better candidates for generating an RBC surface antigen that is essential for invasion. * These results are for *P. maniculatus* because neither study explicitly described the examination of subspecies “*bairdii*”.

Each NHH had at least 26,000 gene entries in the NCBI database, providing evidence that their genes have been well characterized. All 28 NHH lacked orthologs for 5 hBG system genes: *C4B*, *FUT3*, *GYPB*, *GYPE*, and *RHD* (Figure 1). NHH had 4–27 orthologs for 24 hBG system genes: *ABCC1*, *XK*, *FUT1*, *SLC29A1*, *ART4*, *CD59*, *KEL*, *GBGT1*, *SMIM1*, *B4GALNT2*, *CD36*, *CD55*, *ICAM4*, *GYPA*, *SLC14A1*, *CD99*, *FUT2*, *GCNT2*, *ABCG2*, *XG*, *ABO*, *RHCE*, *C4A*, and *CR1*. Recently, CR1 has been linked to *Bm* invasion of human RBCs in vitro [48]. Specifically, IgM against *Bm* Surface Antigen 1 (BmSA1) mediates C3b complement deposition onto *Bm*, which can bind to CR1 on human RBCs, leading to invasion [48]. Orthologs of *CR1* were found in 4/28 NHH. 

All 28 NHH had orthologs for the remaining 22 hBG system genes. Three of these genes encode intracellular enzymes that are not RBC surface proteins: *A4GALT*, *B3GALNT1*, and *PIGG*. Nineteen of these genes encode RBC surface proteins: *ABCB6*, *ABCC4*, *ACHE*, *ACKR1*, *AQP1*, *AQP3*, *BCAM*, *BSG*, *CD151*, *CD44*, *EMP3*, *ERMAP*, *GYPC*, *PIEZO1*, *PRNP*, *RHAG*, *SEMA7A*, *SLC44A2*, and *SLC4A1*. These are promising candidates for generating an RBC antigen that is essential for *Bm* invasion of RBCs.

The literature review revealed several animals are resistant to *Bm*. Although WTD are bitten by *I. scapularis* ticks, which can harbor *Bm*, this species is resistant to experimental *Bm* infection [49]. One study found that chickens, goats, pigs, and dogs could not be experimentally infected with *Bm* [50]. The study exposed 3-month-old “Berger’s dogs” to *Bm* strain ATCCR PRA-99^TM^, and PCR could not detect the parasite 5–55 days after exposure. However, other studies have shown natural infection of *Bm* in dogs and experimental infection of one-year-old beagles [35,51,52]. The reason why some dogs may be resistant to *Bm* is unclear but it could be due to genetic differences in *Bm* strain and/or dogs. 

**Table 1:** Studies showing *Bm* susceptibility of 28 mammals, each of which has a hBG system gene ortholog.

Species	Genbank Common Name	NCBI BLAST Name	NCBI Gene Entries	# Studies Showing Infection *
*Peromyscus leucopus*	white-footed mouse	rodents	32,259	5 [7,8,45,46,53]
*Sorex araneus*	European shrew	insectivores	28,675	5 [37,54,55,56,57]
*Myodes glareolus*	bank vole	rodents	30,720	5 [37,58,59,60,61]
*Mesocricetus auratus*	golden hamster	rodents	36,157	5 [62,63,64,65,66]
*Mus musculus*	house mouse	rodents	107,992	5 [67,68,69,70,71]
*Meriones unguiculatus*	Mongolian gerbil	rodents	34,732	5 [72,73,74,75,76]
*Rattus norvegicus*	Norway rat	rodents	47,827	5 [67,70,77,78,79]
*Felis catus*	domestic cat	carnivores	39,395	5 [51,80,81,82,83]
*Macaca mulatta*	Rhesus monkey	primates	40,413	4 [65,84,85,86]
*Camelus dromedarius*	Arabian camel	even-toed ungulates	37,476	3 [87,88,89]
*Apodemus sylvaticus*	European woodmouse	rodents	34,663	3 [58,59,60]
*Canis lupus familiaris*	dog	carnivores	50,757	3 [35,51,52]
*Arvicola amphibius*	Eurasian water vole	rodents	28,375	2 [90,91]
*Peromyscus maniculatus bairdii*	prairie deer mouse	rodents	36,461	2 [45,46] ^
*Macaca fascicularis*	crab-eating macaque	primates	35,716	2 [84,92]
*Cricetulus griseus*	Chinese hamster	rodents	34,824	2 [38,39]
*Sciurus carolinensis*	gray squirrel	rodents	37,368	2 [7,8]
*Papio anubis*	olive baboon	primates	39,330	2 [36,93]
*Rattus rattus*	black rat	rodents	32,124	2 [94,95]
*Panthera leo*	lion	carnivores	32,109	2 [80,96]
*Mus caroli*	Ryukyu mouse	rodents	32,457	2 [79,94]
*Microtus ochrogaster*	prairie vole	rodents	26,434	1 [97]
*Ursus americanus*	American black bear	carnivores	28,897	1 [98]
*Mus pahari*	shrew mouse	rodents	29,520	1 [94]
*Microtus fortis*	reed vole	rodents	29,738	1 [99]
*Meles meles*	Eurasian badger	carnivores	31,234	1 [100]
*Panthera tigris*	tiger	carnivores	33,598	1 [80]
*Vulpes vulpes*	red fox	carnivores	29,062	1 [101]

* After 5 studies describing *Bm* infection were identified, additional articles were not counted. ^ Neither study explicitly described the examination of the subspecies “*bairdii*”.

## 4. Discussion

The goal of this study was to identify hBG system gene orthologs that are shared by all *Bm* hosts because one of these genes may generate an RBC surface antigen, which is essential for invasion. For 29/51 (56.9%) hBG system genes, at least 1 NHH lacked an ortholog, meaning that these genes are unlikely to generate an RBC antigen that is essential for invasion. For example, *RhD* and *ABO* gene orthologs were found in 0 and 15 of the 28 NHH, respectively. Because persons with blood types A, B, AB, O, RhD-, and RhD+ can be infected with *Bm* [102], a few of our current study’s predictions are correct. For 22/51 (43.1%) hBG system genes, all 28 *Bm* NHH had orthologs, meaning that these genes may generate an RBC antigen which is required for *Bm* invasion. 

There are many limitations of this study. *Bm* may not use an hBG system gene ortholog to invade and may not use the same receptor to invade RBCs from different species. NCBI Gene has not characterized all genes for all *Bm*-susceptible animals. For example, it only lists ~4000 genes for *Bm*-susceptible raccoons (*Procyon lotor*) and no *Bm*-susceptible bird had at least one hBG system gene ortholog, while other birds did [7]. It is possible that we did not identify all NHH from the list of more than 600 animals during the literature review. An abstract from a study on a third-party website translated from Chinese to English was included in our analysis [99]. It was the only study showing *Microtus fortis* is susceptible to *Bm* infection. PCR was not used to confirm infection in all studies. Experimental infection is the definitive method for testing *Bm* susceptibility, but most studies look for natural infection. Only the NCBI database was used to determine orthologs, and it may not have completed its calculations for the 51 genes in the 28 NHH. An animal may have an hBG system gene ortholog, but RBCs may not express the gene product. There is no guarantee that any of the 22 hBG system gene orthologs shared by all NHH are used by *Bm* for invasion. Gorillas have an ortholog of human *BSG* but are not infected with *Pf*, and marmosets are resistant to SARS-CoV-2 infection despite their ACE2 having 92–93% identity to the human form (and being an ortholog) [25]. No experiments were performed to determine whether hBG system antigens are needed for *Bm* invasion.

Another limitation is that *Bm* strains were not documented in this analysis. The categorization of *Bm* can vary as strains are later considered to be distinct species. For example, a parasite formerly known as *Bm*-like is a separate species called *B. vulpes* [103]. One classification describes *Bm sensu stricto* as the US type and categorizes it in Clade 1 [42]. The *Bm* Kobe strain, which was identified in Japan, is in Clade 4. *Bm* Hobetsu and Otsu, identified in China, are genetically the same and listed in Clade 5. A different study found a Midwest and Northeast lineage of *Bm*, with three subpopulations in the latter [104]. It is possible that distinct *Bm* strains have different RBC invasion pathways. However, a monoclonal antibody against basigin blocked *Pf* invasion of RBCs for nine culture-adapted strains and six newly isolated strains [105].

If this study is repeated, it may yield different results. This is because NCBI databases are dynamic. NCBI ortholog calculations could change over time and additional species and genes are continuously being added. In addition, the NCBI website gives slightly different results than the NCBI command-line tools. Importantly, we followed the advice of the NLM Help Desk to use the command-line tools to extract NCBI orthologs. 

The list of 22 hBG system gene orthologs found in all *Bm* NHH can probably be narrowed down because pathogens usually require cell surface proteins on host cells for invasion. To invade RBCs, *Pf* requires basigin, a single transmembrane domain RBC surface protein, and CD55, a GPI-anchored protein. Likewise, *Pk* requires ACKR1, a seven-transmembrane domain RBC surface protein. Of note, orthologs for both genes are present in all 28 *Bm* NHH. SARS-CoV-2 binds to ACE2, while EBV binds to CD21, and HIV binds to CD4, CCR5, and CXCR4, all of which are cell surface proteins. Thus, it is likely that *Bm* also requires a host cell surface protein for invasion. By excluding genes encoding intracellular enzymes, it reduces the list of best gene candidates from 22 to 19. 

The importance of a gene for *Bm* invasion can be determined experimentally. A continuous in vitro culture of *Bm* in human RBCs has recently been reported [48]. So, in vitro invasion assays could be conducted with and without antibodies targeted against specific RBC antigens to determine their importance [48,106]. Alternatively, there are commercially available transgenic mice (*Mus* musculus) with gene knockouts (e.g., *CD44*, *ACHE*, *SEMA7A*, and *SLC4A1*) and gene disruptions (e.g., *PRNP*) that could be used for experimental *Bm* infection. Some mouse strains that are not commercially available have been created by researchers who may be willing to collaborate.

Alternatively, studying hBG system genes in *Bm* patients could provide new clues as to which RBC antigens are important for invasion and disease severity. Polymorphisms of hBG system genes have been well-characterized [30]. This is because transfusing a patient with RBCs expressing an antigen having a single glycan or amino acid difference can result in alloimmunization [107,108,109,110,111,112,113], which can cause a potentially fatal hemolytic transfusion reaction [114,115]. Polymorphisms in the *ABO* gene are responsible for the major blood types of A, B, AB, and O. For RhD, most individuals strongly or weakly express the protein, some express part of the protein, and others lack expression [116]. Only ABO and RhD blood types have been evaluated in *Bm*-infected persons. RhD- American Red Cross blood donors are more likely to have *Bm* infection and RhD- *Bm*-infected patients at Yale and Stony Brook Hospitals were found to have higher peak parasitemia percentages than those who were RhD+ [102,117,118]. Thus, hBG system genes likely impact *Bm*, but our exploration of this topic is just beginning. 

## 5. Conclusions

In summary, this study found 22/51 (43.1%) hBG system genes, 19 (37.3%) of which encode RBC surface proteins, which are expressed in 28/28 *Bm* NHH. One of these genes may generate an RBC surface antigen, which is essential for *Bm* invasion. In vitro, cultures of *Bm* may reveal clues as to which RBC antigens are essential for invasion. If a transgenic mouse strain with a knockout of an hBG system gene is resistant to *Bm* infection, then the gene may generate an antigen essential for invasion. However, knockout mice may be not readily available for orthologs of all of these genes. Studying hBG system genes in *Bm* patients may provide clues as to which RBC antigens are important for disease severity and invasion. Ultimately, if there is an essential RBC receptor for *Bm* invasion, it is likely conserved across mammalian and avian species, even if it is not a hBG system gene. 

## Data Availability

The data presented in this study are available in the Supplementary File.

## Supplementary Materials

The following supporting information can be downloaded at https://www.mdpi.com/article/10.3390/tropicalmed9090195/s1.
